# Synthesis and Evaluation of Antileishmanial and Cytotoxic Activity of Benzothiopyrane Derivatives

**DOI:** 10.3390/molecules25040800

**Published:** 2020-02-12

**Authors:** Cristian Ortiz, Fernando Echeverri, Sara Robledo, Daniela Lanari, Massimo Curini, Wiston Quiñones, Esteban Vargas

**Affiliations:** 1 Química Orgánica de Productos Naturales, Instituto de Química, Facultad de Ciencias Exactas y Naturales, Universidad de Antioquia, calle 70, No. 52–21, Medellín A. A 1226, Columbia; cristian.ortizb@udea.edu.co (C.O.); fernando.echeverri@udea.edu.co (F.E.); 2 PECET-Facultad de Medicina, Universidad de Antioquia, calle 70 No. 52–21, Medellín A. A 1226, Columbia; sara.robledo@udea.edu.co; 3 Dipartimento di Scienze Farmaceutiche, Università di Perugia, Via del Liceo, 1, 06123 Perugia, Italy; daniela.lanari@unipg.it (D.L.); massimo.curini@unipg.it (M.C.)

**Keywords:** *Leishmania*, thiochromenes, benzothiopyrans, thiochroman-4-ones, synthesis, leishmaniasis, cytotoxicity

## Abstract

In continuation of our efforts to identify promising antileishmanial agents based on the chroman scaffold, we synthesized several substituted *2H*-thiochroman derivatives, including thiochromenes, thichromanones and hydrazones substituted in C-2 or C-3 with carbonyl or carboxyl groups. Thirty-two compounds were thus obtained, characterized, and evaluated against intracellular amastigotes of *Leishmania (V)* *panamensis*. Twelve compounds were active, with EC_50_ values lower than 40 µM, but only four compounds displayed the highest antileishmanial activity, with EC_50_ values below 10 µM; these all compounds possess a good Selectivity Index > 2.6. Although two active compounds were thiochromenes, a clear structure-activity relationship was not detected since each active compound has a different substitution pattern.

## 1. Introduction

Cutaneous leishmaniasis (CL) is a group of skin infections caused by intracellular protozoa belonging to the genus *Leishmania* and transmitted by *Lutzomyia* and *Phlebotomus* sand flies. CL is a poverty-associated disease considered by the World Health Organization as one of the 17 neglected diseases due to a lack of interest by the pharmaceutical industry to develop new drugs and a wide distribution around the world. More than 310 million people at risk, and about one million new cases are occurring annually. CL cases occur mainly in Afghanistan, Algeria, Brazil, Colombia, Iran, and Syria [1].

To date, a few drugs are available against leishmaniasis, including pentavalent antimonials, amphotericin B, and miltefosine. However, existing treatments are unsatisfactory because of their high cost, toxicity, need for prolonged treatment, and reduced efficacy, therefore, there is an urgent need to develop new molecules with novel modes of action against this disease.

The chroman (benzopyran) moiety is considered a privileged scaffold in medicinal chemistry since it exhibits a broad range of biological activities such as antiemetic, anti-hypertensive, anti-malarial, and insecticidal activities, among others [2,3,4,5]. From a bioisosteric point of view, replacement of the oxygen atom (O) by a sulfur atom (S) it may cause significant changes in the pharmacology activity since the changes in electron-donating potential and the size of sulfur cause solubility [6], polarity, hydrogen bonding and metabolism [7] modifications. In a previous paper, we have reported that acyl hydrazone derivatives of chromanone and thiochromanone have good in vivo antileishmanial activity with effective concentration (EC_50_) values of 20.1 and 25.0 µM respectively, in addition to anti-inflammatory and wound-healing properties [8]. Besides, acyl hydrazone derivatives of thiochroman-4-ones have been reported as potent inhibitors of cysteine proteases which has implications in the treatment of Chagas’ disease [9,10]. A SAR study involving thiochroman-4-one derivatives found that compounds containing 1,1-dioxo-2-aryl-4*H*-thiochromen-4-one scaffolds displayed good antileishmanial activity below 10 µM. Ester and amide derivatives of thiochroman-4-ones were also include in this study; the amides tend to form intermolecular and intramolecular hydrogen bonds with other groups [11] and esters are used for masking polar moieties and taking advantage of the availability of endogenous, non-specific esterases, release the molecule inside the cell [12].

Herein, we report the synthesis of a library of compounds bearing a benzothiopyran moiety, including thiochromenes, thiochromanones and acyl hydrazones with a combination of fragments possessing a substituted carbonyl group in C-2 or C-3 and their in vitro anti-leishmanial and cytotoxic activities.

## 2. Results

### 2.1. Synthesis, Dehydration and Oxidation of Thiochromanols

In our search for methods for the synthesis of *2H*-thiochromene-like compounds, we first explored the synthetic methodologies used for their oxygenated counterparts. Several methodologies have been proposed, which include, among others, the Petasis reaction [13] of salicylaldehydes, tandem Michael additions [14], PPh_3_-catalyzed domino reactions [15] for the synthesis of chroman derivatives and the domino oxa-Michael aldol reaction [16]. The latter approach seemed likely to succeed with the mercapto counterpart.

**Scheme 1:**
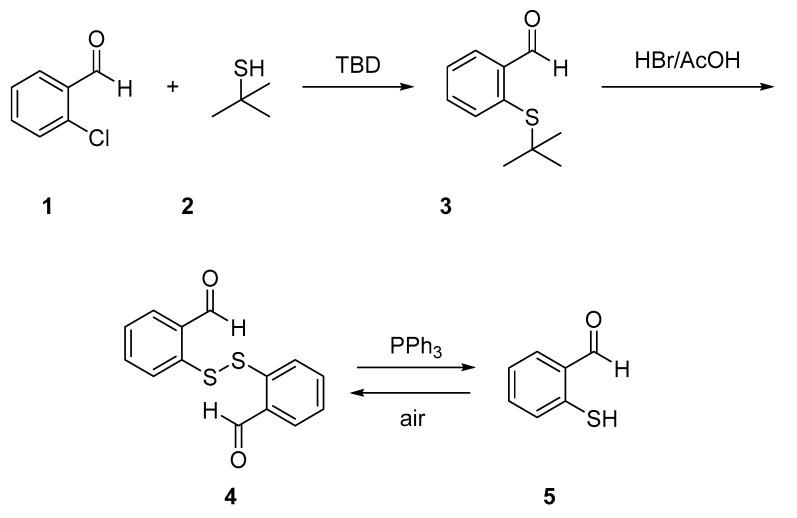
Synthesis of 2-mercaptobenzaldehyde.

Synthesis of the precursor 2-mercaptobenzaldehyde (mercaptosalicylaldehyde, Scheme 1) started from 2-chlorobenzaldehyde (**1**) and *tert*-butylthiol (**2**), following a modification of the Chemburkar procedure [17] using 1,5,7-triazabicyclo[4.4.0]dec-5-ene (TBD) under solvent-free conditions leading to high yields of the desired 2-(*tert*-butylthio)benzaldehyde (**3**). Treatment of **3** with a mixture of HBr/acetic acid in dimethyl sulfoxide (DMSO) gave the 2,2’-dithiodibenzaldehyde (**4**). Following the work of Humphrey and Hawkins [18], compound **4** was next allowed to react with PPh_3_ to give 2-mercaptobenzaldehyde (**5**), which readily oxidizes in air to give again disulfide **4**.

**Scheme 2:**
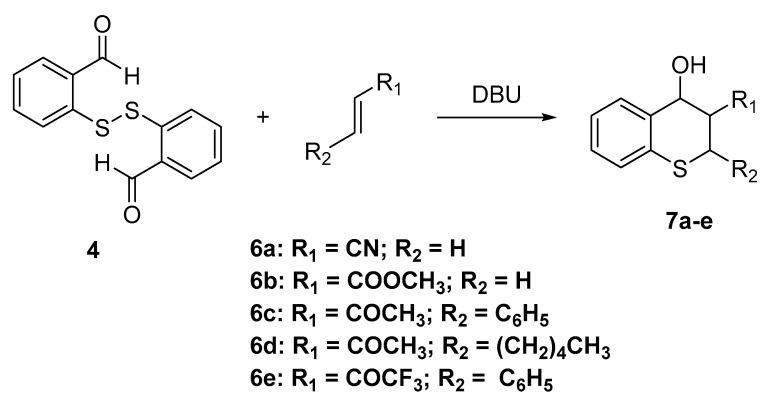
Synthesis of 2*H*-thiochromanols derivatives **7a**–**e**.

Considering the tedious procedure to obtain **5** and its relatively easy oxidation in the presence of air, we decided to explore the synthesis of thiochromene compounds directly from aldehydes **3** or **4**. Thus, disulfide **4** was allowed to react with activated alkenes **6a**–**e** in the presence of 1,8-diazabicyclo[5.4.0]undec-7-ene (DBU) and triphenylphosphine, whereby thin layer chromatography (TLC) of the reaction mixture showed various spots with similar Rf values. ^1^H-NMR analysis of these crude mixtures revealed that this set of spots corresponds to the stereoisomers of thiochromanols **7a**–**e** (Scheme 2). Due to the difficulty in separating the mixture of stereoisomers and the inconvenience of using these mixtures in biological tests, we decided to use these compounds as a mixture in the following reactions. The reaction crude was passed through a chromatography column to remove the DBU and PPh_3_ and the excess of the starting materials.

**Scheme 3:**
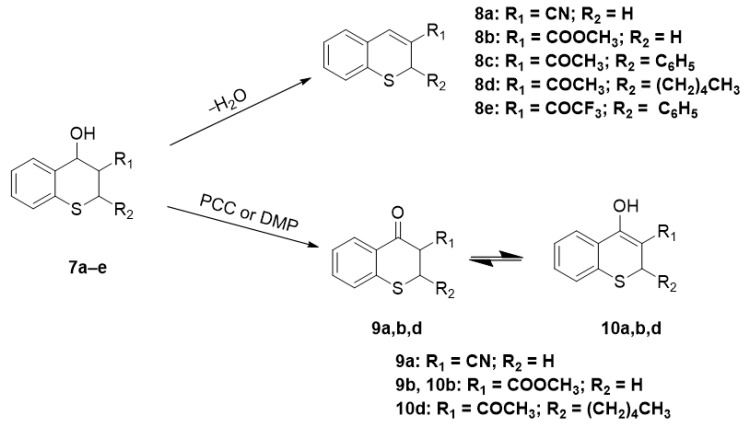
Synthesis of 2*H*-thiochromenes **8a**–**e**, ketones **9a,b,d** and the enol forms **10a,b,d**.

To obtain *2H*-thiochromenes **8a**–**e** (Scheme 3), the mixtures of diastereomeric thiochromanols **7a**–**e** were dehydrated by heating in the presence of acid (Amberlyst 15 or HCl) or even by simple direct heating of the mixture containing the DBU and PPh_3_.

In the search for compounds bearing a thiochroman-4-one moiety, thiochromanols **7a**–**e** were treated with oxidants like pyridinium chlorochromate (PCC) or Dess-Martin Periodinane reagent (DMP, Scheme 3). Oxidation of **7c** and **7e** did not give the expected products**.** On the other hand, compounds **7a**,**b**,**d** gave oxidation products which could tautomerize and were analyzed by NMR to determine in which tautomeric form they exist.

NMR spectra of the oxidation product of **7a** showed only one set of signals in which the protons at δ 4.18 (dd, *J* = 11.6, 3.7 Hz, 1H), 3.67 (dd, *J* = 13.5, 11.7 Hz, 1H) and 3.57 − 3.40 (m, 1H) and the ^13^C- NMR δ 184.6 characteristic of a ketone group indicate that the compound exists only as its keto form **9a**.

In the case of the oxidation of **7b**, the NMR spectra showed two sets of signals, indicating the existence of two tautomeric forms. In the keto form **9b**, proton H5 appears as a doublet at 8.17 ppm, *J* = 7.9 Hz, while in the enol form **10b** this proton signal is shifted at 7.89, d, *J* = 7.7 Hz, both signals integrating for 0.2 and 1.0, respectively, the same ratio was can be calculated with the protons of the methoxy group of the ester. A calculation of peak-intensity showed that this tautomeric mixture consisted of 83% of the enol form and 17% of the keto form.

A similar analysis for the oxidation **7d** showed no proton at C3, and the ^13^C-NMR resonance at δ 195.4 and 174.0 is not consistent with a diketo compound **9d**, which indicates that it exists only as an enol compound **10d.** It can be assumed that this enol form was stabilized by the intramolecular hydrogen bond.

### 2.2. Alternative Synthesis of 2H-Thiochromene Compounds

**Scheme 4:**
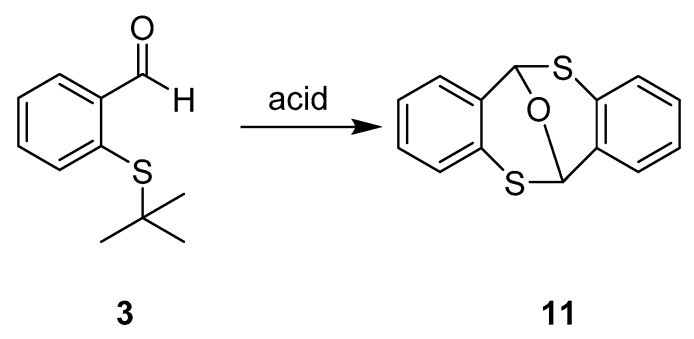
Proposed structure of the dimeric hemithioacetal **11**.

Taking into account that S-*tert*-butyl groups are cleaved under acidic conditions [19,20], treatment of compound **3** with hydrochloric acid (HCl) or *p*-toluenesulfonic acid (PTSA) gave a dimeric hemithioacetal **11** (Scheme 4), previously reported by Dickmann [21]. Formation of compound **11** must happen through the formation of 2-mercaptobenzaldehyde (**5**), followed by dimerization.

**Scheme 5:**
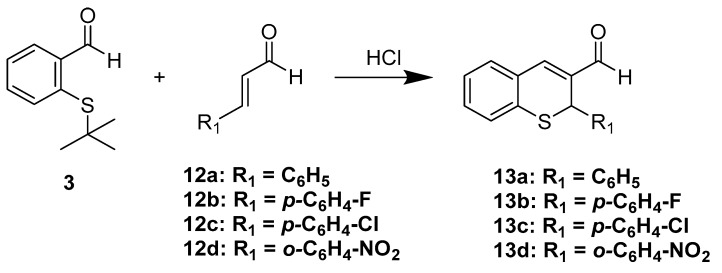
Direct synthesis of 2*H*-thiochromenes starting from **3** and cinnamic aldehydes.

The above results led us to think that compound **5** formed in situ could react with suitably activated alkenes to yield the addition product; thus, we carried out the reaction of **3** in the presence of cinnamaldehydes **12a**–**d** using 12M HCl. Thus, substituted 2-phenyl-2*H*-thiochromene-3-carbaldehydes **13a**–**d** in moderate to good yields were effectively produced (Scheme 5).

**Scheme 6:**
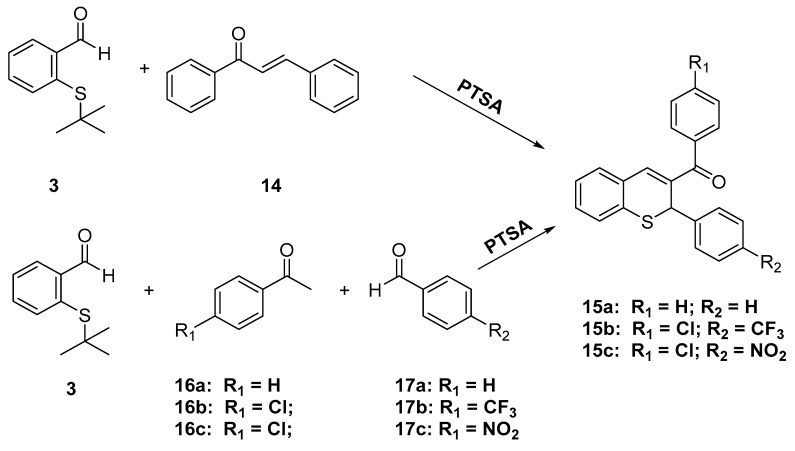
Synthesis of (aryl-2*H*-thiochromen-3-yl)-methanones from chalcones.

Similarly, *trans*-chalcone reacted with **3** in the presence of 4-toluenesulfonic acid in toluene to give phenyl-(2-phenyl-2*H*-thiochromen-3-yl)-methanone (**15a**) in 46% yield (Scheme 6). Acid-catalyzed in situ formation of the chalcones followed by reaction with 2-(*tert*-butylthio)-benzaldehyde in a one-pot procedure also resulted in the formation of the desired aryl-2*H*-thiochromen-methanones **15b**–**c.**

### 2.3. Synthesis of 4-oxo-thiochroman-2-Carboxylic Acid and Its Derivatives

**Scheme 7:**
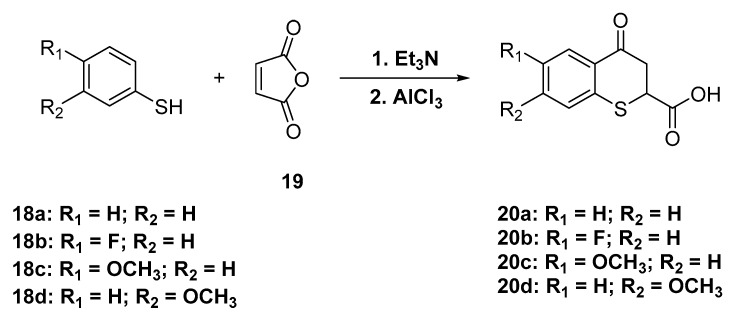
Synthesis of 4-oxothiochroman-2-carboxylic acid.

Derivatives of thiochroman-4-one are typically prepared by conjugate additions of thiophenols to α,β-unsaturated carboxylic acids followed by treatment with a strong dehydrating agent or by formation of the corresponding acid chlorides followed by treatment with a Lewis acid like AlCl_3_ or SnCl_4_. Similarly 4-oxo-thiochroman-2-carboxylic acids **20a**–**d** were obtained by reacting thiophenols **18a**–**d** with furan-2,5-dione (maleic anhydride, **19**) in the presence of triethylamine and with subsequent treatment with AlCl_3_ (Scheme 7).

In order to get a set of compounds that allow us to perform a SAR analysis to establish the influence of the functional groups and the length of the side chains we prepared a set of esters and amides of 4-oxo-thiochroman-2-carboxylic acids.

**Scheme 8:**
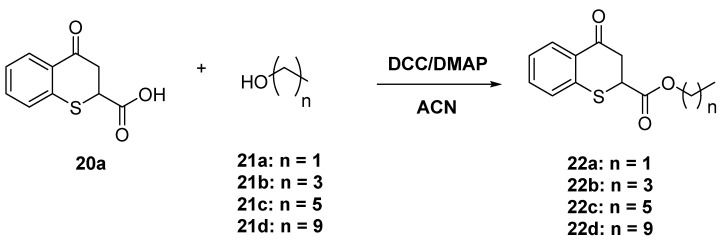
Esters of 4-oxo-thiochroman-2-carboxylic acid.

Esters of 4-oxo-thiochroman-2-carboxylic acid **22a** were prepared by reaction with the corresponding alcohols **21a**–**d** using dicyclohexylcarbodiimide (DCC) as a coupling agent and 4-dimethylaminopyridine (DMAP) as a catalyst (Scheme 8).

**Scheme 9:**
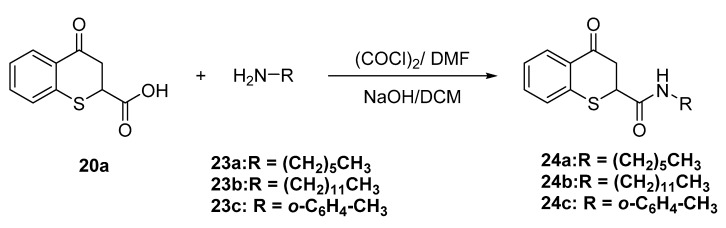
Synthesis of amides of 4-oxo-thiochroman-2-carboxylic acid.

Amides **24a**–**c** were prepared by reacting the corresponding amines **23a**–**c** with 4-oxo-thiochroman-2-carboxylic acid **20a** under Schotten-Baumann conditions (Scheme 9) using a biphasic system containing in the organic layer, oxalyl chloride (COCl)_2_ and dimethyl formamide as catalyst (DMF) and aqueous sodium hydroxide.

### 2.4. Synthesis of Acyl Hydrazone Derivatives

**Scheme 10:**
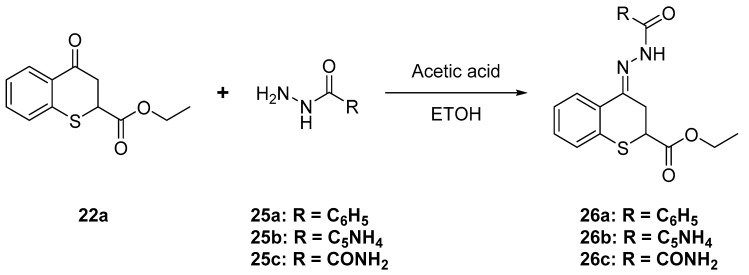
Synthesis of acyl hydrazine derivatives.

As it was mentioned before, compounds having an acyl hydrazone moiety have shown good antiparasitic activities. According to this we decided to prepare some derivatives with this structural feature. Acyl hydrazone derivatives **26a**–**c** were prepared by reaction of ethyl 4-oxothiochromane-2-carboxylate with acyl hydrazides **25a**–**c** in the presence of acetic acid (Scheme 10).

### 2.5. Resolution of Enantiomers of 4-oxo-thiochroman-2-carboxylic Acid Using Brucine

**Scheme 11:**
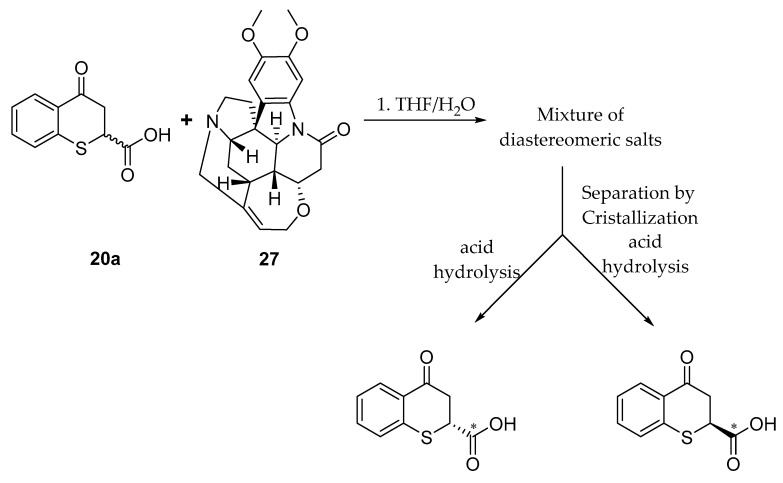
Chiral resolution of 4-oxothiochroman-2-carboxylic acid **20a**.

To establish the difference in antileishmanial activity of the enantiomers of 4-oxo-thiochroman-2-carboxylic acid (**20a**) we attempted the chiral resolution by diastereomeric salt formation through reaction with optically pure (−) -brucine **27** as a chiral selector (Scheme 11). Thus, **20a** was allowed to react with (−) -brucine **27** in a mixture of THF/water 4:1 at room temperature overnight, to yield a mixture of diastereomeric salts. Attempts were made to crystallize the mixture by slow evaporation of different solvents (acetone, THF, water, ethanol and ethyl acetate). Finally, crystalline salts were obtained after evaporation of ethyl acetate. Crystals were separated from mother liquors and were subjected to acid hydrolysis to yield the salt of (−) – brucine and an enantiomerically enriched sample of 4-oxo-thiochroman-2-carboxylic acid (+)-**20a** with specific rotation [α]^25^_D_ = +35.6. After evaporating the ethyl acetate from mother liquors, we obtained an enriched sample of the less crystalline diastereoisomeric salt. Acid hydrolysis of the residue of the mother liquors yield a mixture of enantiomers with specific rotation of [α]^25^_D_ = −10.8 for (−)-**20a.** We did not determine absolute configuration nor enantiomeric excess.

**Scheme 12:**
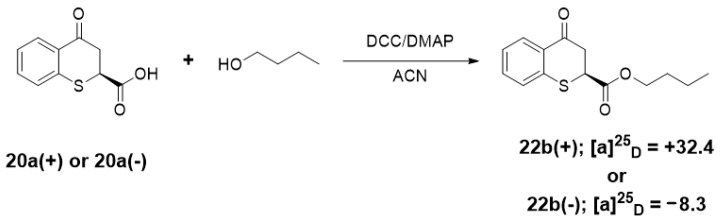
Enantiomerically enriched butyl 4-oxothiochromane-2-carboxylates.

Enantiomerically enriched samples of butyl 4-oxothiochromane-2-carboxylates (+)-**22b** with ${\left[\alpha \right]}_{D}^{25}$ = +32.4 and (−)^-^**22b** ${\left[\alpha \right]}_{D}^{25}$ = −8.3, respectively, were obtained by reaction of butanol with enantiomerically enriched samples of 4-oxo-thiochroman-2-carboxylic acid (+)-**20a** or (−)-**20a** (Scheme 12).

### 2.6. Antileishmanial and Cytotoxic Activities

The synthesized compounds were evaluated for their in vitro antileishmanial and cytotoxic activities (Table 1), following the methods of Pulido et al. [22] Amphotericin B, with EC_50_ and LC_50_ values of 0.05 µM and 56.8 µM, respectively, was used as a control.

## 3. Discussion

Leishmaniasis is a disease that threatens and affects almost 20% of the world’s population, but, there are very few effective drugs available for its treatment, so new molecules are urgently needed. However, this task is a priority for the pharmaceutical industry, so the affected countries must seek their own alternatives. Recently we reported the in vivo leishmanicidal activities of compounds bearing a benzothiopyran scaffold and also the acyl hydrazones derived from chroman and thiochromans [8,23]. To optimize their effects, in this work we prepared thirty-two compounds, including thiochromenes, thichroman-4-ones and acyl hydrazones substituted in C-2 or C-3 with carbonyl/nitrile or carboxyl groups.

The compounds can be grouped into three types, according to their activity. The most active were those with EC_50_ < 20 µM, those with marginal activity had an EC_50_ between 20–40 µM and the inactive compounds have EC_50_ > 40. The most active compounds include three the thiochromenes **8a**, **8d**, **13d** and compound **10d**. Substituents at C-2 and C-3 in the former compounds do not have a structural relationship between them. Compound **8a** with a nitrile at C-3 is the most active, but, surprisingly **8b** with a methyl ester is practically inactive, although the methyl ester is considered a nitrile isostere [24].

Compounds **8d** and **10d** with an alkyl chain at C-2 showed high leishmanicidal activity, and changing the alkyl chain for a phenyl group resulted in a decrease in anti-leishmanial activity with an increase in the cytotoxicity. Although none of the compounds bearing a phenyl or aryl group at C-2 showed high anti-leishmanial activity most of the 2-arylthiochromenes displayed a marginal activity, indicating promising compounds for further optimization. On the other hand, if the C-3 methyl ketone is substituted by phenyl or aryl groups, the cytotoxicity decreases while maintaining marginal activities between 37.9–43.9 µM.

Thiochromenes of type C-3-formyl, **13a**,**b**,**c** with aryl groups in C-2 have a range of activity similar to compounds **8e** and **15a**,**b**,**c** all having electron-withdrawing groups. The above seems to indicate that steric factors and pi interactions are involved in the interaction of these compounds with a putative receptor of *Leishmania* amastigote.

On the other hand, the importance of a C-3 carbonyl substituent is demonstrated in the series of compounds **20a**–**d**, **22a**–**d** and **24a**–**c** all bearing the thiochroman-4-one scaffold without substituents at C-3, which were practically inactive. The previous esters and amides were prepared thinking of performing a SAR study to analyze the effect of chain size on leishmanicidal and cytotoxic activity, but all these compounds showed very low activity.

Surprisingly, two acyl hydrazones **26a** and **26b** showed a marginal activity EC**_50_** 28.5 and 53.2 µM, unlike the compounds that we have previously analyzed [8], which seems to indicate that the substitution by a C-2 carbonyl of a thiochroman-4-one interferes with its mechanism of action against amastigotes of *L. panamensis* parasites**.**

In conclusion, in search of new chemotherapeutic agents against leishmaniasis based on the benzothiopyran moiety, we synthesized thirty-two structurally diverse compounds. These compounds possess relevant in vitro antileishmanial activity with moderate cytotoxicity. Compounds **8d** and **10d**, which differ only in the hydroxy group at C-4, were the most promising compounds among this library with good antiparasitic activity and Selectivity Index. Biological studies with *Leishmania* parasite enzymes need to be performed to identify the potential targets. Structural modification on ring A of **8d** and **9d**, **10d** could enhance the antileishmanial activity or reduce cytotoxicity. Overall, **8d**, **9d**, and **10d** represents potential hits in the search for new chemotherapeutic agents for the treatment of leishmaniasis.

## 4. Materials and Methods

### 4.1. Chemistry

#### 4.1.1. General Information

All commercially available reagents and solvents were obtained from commercial suppliers and used without further purification. The reaction progress was monitored with thin layer chromatography on silica gel TLC aluminum sheets (60F_254,_ Merck, Darmstadt, Germany). The melting points were determined using a Mel-Temp apparatus (Electrothermal, Staffordshire, UK) and are uncorrected. FTIR spectra were obtained on a Bruker Alpha FTIR spectrometer (Bruker Optic GmbH, Ettlingen, Germany). ^1^H and ^13^C nuclear magnetic resonance (NMR) spectra were recorded using Bruker 300 and 400 spectrometers (300 and 400 MHz for 1H, 75, and 100 MHz for ^13^C). Chemical shifts were reported relative to internal tetramethyl silane (δ 0.00 ppm) for ^1^H, and CDCl_3_ (δ 77.0 ppm) for ^13^C. HRMS was obtained using Q-TOF quadrupole/orthogonal mass spectrometry (Waters, Milford, MA, USA) in positive mode (reported as [M + H]^+^ or [M + Na]^+^ ).

#### 4.1.2. Synthesis of the Benzothiopyranes and Derivatives

##### *2-(tert-Butylthio)benzaldehyde* (**3**) 

In a screw-capped flask equipped with a stir bar, DMSO (2.0 mL, 0.028 mol, 4 equivalent) and potassium hydroxide (KOH; 470 mg, 8.4 mmol,1.1 equivalent) were mixed under nitrogen and stirred at room temperature. After 30 min, the KOH was crushed with a spatula, and 2-methyl-2-propanethiol (610 µL, 0.0106 mol, 1.5 eq) was added, and the mixture was stirred for 20 min. 2-Chlorobenzaldehyde (810 μL, 7.2 mmol, 1.0 equivalent) was added, and the reaction was heated to 110 °C for 90 min. After that, the reaction mixture was diluted with water (20 mL) and extracted with ethyl acetate (2 × 30 mL). The combined organic layers were washed with water (30 mL) and brine (30 mL), dried (anhydrous Na_2_SO_4_), and concentrated on a rotary evaporator the residue was purified by chromatography using a mixture of 10% ethyl acetate in hexanes to afford 2-(*tert*-butylthio)benzaldehyde **15** (980 mg, 70% yield) along with some unreacted 2-chlorobenzaldehyde. ^1^H-NMR (300 MHz, CDCl_3_) 10.78 (s, 1H), 7.98 (d, *J* = 4.3 Hz, 1H), 7.62–7.50 (m, 1H), 7.45–7.30 (m, 1H), 1.30 (s, 9H). GC-MS: *m*/*z* (%) = 194 (27) [M^+^], 161 (8), 138 (100), 77 (5).

##### *2,2′-Disulfanediyldibenzaldehyde* (**4**)

A 25-mL round-bottom flask containing 2-(*tert*-butylthio)benzaldehyde (**3**, 194 mg, 1.0 mmol, 1 equivalent) was placed in an ice bath. Acetic acid (343 μL, 6 mmol, 6.0 equivalents), 48% aqueous HBr (343 μL, 3 mmol, 3.0 equivalents) and DMSO (73 μL, 1.0 mmol, 1.0 equivalent) were then added to the resulting cooled mixture. The reaction mixture was then allowed to warm to room temperature and then was stirred overnight. The reaction mixture, containing a solid precipitate, was diluted with cold water (2 mL), filtered, dried and purified by column chromatography using 20% ethyl acetate in hexanes as eluent to afford 2,2-disulfanediyldibenzaldehyde (**4**). Yield 73%. White solid. m.p.:.: 149–151 °C. ^1^H-NMR (300 MHz, CDCl_3_) δ 10.19 (s, 2H), 7.85 (dd, *J* = 7.5, 1.4 Hz, 2H), 7.75 (d, *J* = 7.8 Hz, 2H), 7.47 (td, *J* = 7.7, 1.6 Hz, 2H), 7.37 (dd, *J* = 10.6, 4.2 Hz, 2H). ^13^C-NMR (75 MHz, CDCl_3_) δ 192.1, 140.2, 135.0, 134.5, 134.0, 126.8, 126.5. IR ν: 1664, 1582, 1555. GC-MS: *m*/*z* (%) = 274 (14) [M^+^], 137 (100), 109 (54). HRMS-ESI (*m*/*z*): calcd. for C_14_H_10_O_2_S_2_ [M + Na]^+^ 297.0014, found: 297.0025.

##### *2-Mercaptobenzaldehyde* (**5**)

In a 25 mL round bottom flask, 2,2-disulfanediyldibenzaldehyde (**4**, 177 mg, 0.65 mmol, 1 equivalent), was dissolved in a mixture of DMF (5.4 mL), MeOH (5.4 mL) and of water (3.0 mL; all solvents were deoxygenated before use) then tripheylphosphine (253 mg, 0.975 mmol, 1.5 equivalent) was added, and the mixture stirred a room temperature for 30 min. After that the reaction mixture was diluted with water (20 mL) and extracted with deoxygenated diethyl ether (2 × 30 mL). The combined organic layers were washed with deoxygenated water (30 mL) and brine (30 mL), dried (anhydrous Na_2_SO_4_), and concentrated on a rotary evaporator the residue was purified by chromatography using a mixture of 10% ethyl acetate in hexanes to afford 2-mercaptobenzaldehyde (**5**), in 73% yield. During the whole process, the presence of air in the mixture was avoided to prevent oxidation back to the disulfide **4.** ^1^H-NMR (300 MHz, CDCl_3_) δ 10.02 (s, 1H), 7.73 (dd, *J* = 8.5, 7.7 Hz, 1H), 7.38 – 7.20 (m, 3H), 5.48 (s, 1H).

##### *2H-Thiochromene-3-carbonitrile* (**8a**) 

In a round-bottom flask were placed 2,2-disulfanediyl-dibenzaldehyde (**4**, 137 mg, 0.5 mmol, 1 equivalent), acrylonitrile 58 mg (0.55 mmol, 2.2 equivalents) and DBU 84 mg (0.55 mmol, 1.1 equivalents) under nitrogen, and the mixture was heated with stirring at 80 °C for 24 h, After cooling to room temperature, the crude reaction mixture was then purified by column chromatography over silica gel using hexane/EtOAc (5:1) as eluent to afford **7a** as a white solid (77 mg, 45%). Alternatively, 2*H*-thiochromene-3-carbonitrile (**7a**) was prepared by a different methodology, as follows: 

*Step 1*. In a screw-capped flask, 2,2-disulfanediyl-dibenzaldehyde (**4**, 70 mg, 0.25 mmol, 1 equivalent); triphenylphosphine 131 mg (0.5 mmol, 2 equivalents) were dissolved in THF (5 mL), the mixture was stirred at room temperature under an argon atmosphere. After 20 min an excess of acrylonitrile (80 mg, 1.5 mmol, 6 equivalents) was added and after another 10 min of reaction a TLC plate showed the formation of 2-mercaptobenzaldehyde (**5**) and the mixture of stereoisomers of 4-hydroxy-thiochromane-3-carbonitrile. The reaction mixture is stirred overnight, and after that time, the only products are the 4-hydroxythiochromane-3-carbonitriles.

*Step 2*. The mixture of 4-hydroxythiochromane-3-carbonitriles is heated overnight at reflux in dichloroethane with 20% mol of Amberlyst 15 after that, the solvent was evaporated on a rotary evaporator and diluted in the minimum amount of CH_2_Cl_2_ and purified by column chromatography using a mixture of 20% ethyl acetate in hexanes as eluent to afford 2*H*-thiochromene-3-carbonitrile **8a** in 68% yield (calculated from the dimer). Mp. 96–98 °C. ^1^H-NMR (400 MHz, CDCl_3_) δ 7.30 − 7.19 (m, *J* = 7.5 Hz, 2H), 7.20 – 7.10 (m, 3H), 3.57 (s, 2H). ^13^C-NMR (101 MHz, CDCl_3_) δ 142.4, 132.9, 131.1, 130.3, 130.3, 127.7, 126.5, 118.5, 103.9, 26.0. IR ν: 2207, 1615, 1435, 753. HRMS-ESI (*m*/*z*): calcd. for C_10_H_7_NS [M + H]^+^ 174.0372, found: 174.0380.

##### *Methyl 2H-thiochromene-3-carboxylate* (**8b**)

In a screw-capped flask, 2,2-disulfanediyldibenzaldehyde (**4**, 137 mg, 0.5 mmol, 1 equivalent), methylacrylate (130 mg, 1.5 mmol, 3.0 equivalents) and DBU (230 mg, 1.5 mmol, 3.0 equivalents) were mixed under an argon atmosphere and then heated to 80 °C for 24 h, After cooling to room temperature the reaction mixture was dissolved with CH_2_Cl_2_ and poured directly onto a chromatography column that was eluted using 10% ethyl acetate in hexanes to afford methyl 2*H*-thiochromene-3-carboxylate (**8b**) in 48% yield as a yellowish solid with mp. 34–35 °C. ^1^H-NMR (400 MHz, CDCl_3_) δ 7.55 (s, ^1^H), 7.30 − 7.16 (m, 3H), 7.13 (d, *J* = 7.4 Hz, 3H), 3.84 (s, 3H), 3.73 (s, 2H). ^13^C-NMR (100 MHz, CDCl_3_) δ 166.4, 137.3, 134.0, 131.3, 130.6, 130.2, 127.1, 125.8, 123.0, 52.2, 24.0. IR ν: 2952, 1703, 1434, 1235, 751. HRMS-ESI (*m*/*z*): calcd. for C_11_H_10_O_2_S [M + H]^+^ 207.0474, found: 207.0185.

##### *1-(2-Phenyl-2H-thiochromen-3-yl)ethan-1-one* (**8c**)

In a 10 mL round bottom flask equipped with a reflux condenser were added (*E*)-4-phenylbut-3-en-2-one (146 mg, 1.0 mmol) and 2-(*tert*-butylthio)benzaldehyde (**3**, 250 mg, 1.3 mmol) and to this mixture was added concentrated hydrochloric acid (12M, 2.0 mL) and the mixture was placed in a preheated oil bath at 110 °C and held for two hours. Thereafter water (10 mL) was added and the mixture was extracted with dichloromethane (3 × 15 mL), the combined organic layers were washed with saturated NaHCO_3_ solution. After drying over Na_2_SO_4_, the solvent was evaporated, and the residue was purified by column chromatography to yield 106 mg (40%) of 1-(2-phenyl-2*H*-thio-chromen-3-yl)ethan-1-one **27**, as a yellowish solid. Mp: 115–116 °C. ^1^H-NMR (400 MHz, CDCl_3_) δ 7.67 (s, 1H), 7.38 (d, *J* = 7.8 Hz, 1H), 7.25 (dd, *J* = 3.9, 2.8 Hz, 1H), 7.21 – 7.13 (m, 5H), 5.36 (d, *J* = 1.6 Hz, 1H), 2.49 (s, 3H). ^13^C-NMR (101 MHz, CDCl_3_) δ 196.5, 142.0, 137.4, 134.0, 132.8, 131.1, 130.7, 130.4, 128.5, 127.6, 127.6, 126.5, 125.7, 38.8, 25.6. IR ν: 3031, 1656, 1622, 1196, 900. HRMS-ESI (*m*/*z*): calcd. for C_17_H_14_OS [M + H]^+^ 267.0838, found: 267.0345.

##### *1-(2-Pentyl-2H-thiochromen-3-yl)ethan-1-one* (**8d**) 

In a 10 mL round bottom flask were added 2,2-disulfanediyldibenzaldehyde (**4**, 140 mg, 0.5 mmol), 2-Nonanone (145 mg, 1.0 mmol) and triphenylphosphine (262 mg, 1.0 mmol) dissolved in THF (5 mL) and the mixture was stirred under an argon atmosphere at room temperature overnight. Column chromatography of the crude residue after solvent removal gave 195 mg (70%) of a mixture of stereoisomers of 1-(4-hydroxy-2-pentylthiochroman-3-yl)ethan-1-one. Later 140 mg of the isomeric alcohols was dissolved in THF (5 mL containing Amberlyst 15 (20 % mol) in a 10 mL round bottom flask equipped with a reflux condenser and the mixture was heated to reflux overnight during 20 h. After cooling to room temperature the reaction mixture was dissolved with CH_2_Cl_2_ and the crude was purified by column chromatography over silica gel using hexane/EtOAc (9:1) as eluent to afford 40 mg (29%) of 1-(2-pentyl-2*H*-thiochromen-3-yl)ethan-1-one (**8d**) as a yellowish oil. ^1^H-NMR (400 MHz, CDCl_3_) δ 7.45 (s, 1H), 7.37 (d, *J* = 8.0 Hz, 1H), 7.34 − 7.24 (m, 2H), 7.20 (td, *J* = 7.3, 1.4 Hz, 1H), 4.11 (dd, *J* = 8.0, 5.6 Hz, 1H), 2.52 (s, 3H), 1.47 (dt, *J* = 10.8, 3.7 Hz, 2H), 1.35 − 1.15 (m, 6H), 0.88 (t, *J* = 6.8 Hz, 3H. ^13^C-NMR (75 MHz, CDCl_3_) δ 196.8, 136.4, 133.3, 130.8, 130.6, 128.1, 125.5, 36.2, 34.4, 31.2, 25.7, 25.5, 22.5, 14.1. Note: the signals at 130.6 and 133.3 each correspond to two overlapped peaks. IR ν: 2927, 1635, 1407, 1388, 756. HRMS-ESI (*m*/*z*): calcd. for C_16_H_20_OS [M + H]^+^ 261.1308, found: 261.1320. 

##### *4-Oxo-thiochromane-3-carbonitrile* (**9a**)

According with Step 1 of the procedure for the synthesis of compound **8a** we prepared a mixture of stereoisomers of 4-hydroxythiochromane-3-carbonitrile, then this mixture (191 mg, 1.0 mmol) was dissolved in dichloromethane (5.0 mL) and mixed with the Dess-Martin periodinane reagent (430 mg, 1 mmol). After 1 h of stirring at room temperature the crude mixture was purified by column chromatography over silica gel using hexane/EtOAc (4:1) as eluent to afford the desired 4-oxothiochromane-3-carbonitrile (**9a**, 60 mg, 30%) as a white solid with mp: 79–81°C. ^1^H-NMR (300 MHz, CDCl_3_) δ ^1^H NMR (300 MHz, CDCl_3_) δ 8.17 (dd, *J* = 8.0, 1.4 Hz, 1H), 7.61–7.44 (m, 1H), 7.42–7.21 (m, 2H), 4.18 (dd, *J* = 11.6, 3.7 Hz, 1H), 3.67 (dd, *J* = 13.5, 11.7 Hz, 1H), 3.57–3.40 (m, 1H). ^13^C-NMR (75 MHz, CDCl_3_) δ 184.6, 141.0, 134.7, 130.5, 128.9, 127.7, 126.0, 115.1, 41.8, 29.4. IR ν: 2918, 2256, 1678, 1582, 1430. HRMS-ESI (*m*/*z*): calcd. for C_10_H_7_NOS [M + Na]^+^ 212.0141, found: 212.0153.

##### *Methyl 4-oxo-thiochromane-3-carboxylate* (**9b**, **10b**) 

In a screw-capped flask, 2,2-disulfanediyl-dibenzaldehyde (**4,** 70 mg, 0.25 mmol, 1 equivalent) and triphenylphosphine (131 mg (0.5 mmol, 2 equivalents), were dissolved in THF (5 mL), and the mixture was stirred at room temperature under an argon atmosphere. After 20 min an excess of acrylonitrile (80 mg, 1.5 mmol, 6 equivalents) was added and after another 10 min of reaction a TLC plate showed the formation of 2-mercaptobenzaldehyde (**5**) and a mixture of 3-cyano-4-hydroxy-2*H*-thiochroman isomers. The reaction mixture was stirred overnight, and after that, the only products are the mixture of stereoisomers of 3-cyano-4-hydroxy-2*H*-thiochroman. A portion of the above mixture (112 mg) was dissolved in dichloromethane (5.0 mL) and mixed with the Dess-Martin periodinane reagent (430 mg, 1 mmol). After 1 h of stirring at room temperature, the crude mixture was purified by column chromatography to afford 40 mg (36%) of the desired methyl 4-oxo-thiochromane-3-carboxylate as a white solid with mp: 85–86 °C. Methyl 4-oxo-thiochromane-3-carboxylate (**9b**) exists in solution in equilibria with its tautomeric methyl 4-hydroxy-2*H*-thiochromene-3-carboxylate enol form **10b**. The ^1^H-NMR spectrum in CDCl_3_ revealed the that the ratio of tautomers **9b** and **10b** was 1:5. Proton H5 appears as a doublet at 8.17 ppm, *J* = 7.9 Hz, the proton H5 of the enol form has a chemical shift of 7.89 d, J = 7.7 Hz the integration areas are 0.2 and 1.0 respectively, which indicates that the enol form corresponds to 83% of the mixture. The same ratio can be calculated with the protons of the methoxy group of the ester. ^1^H-NMR (300 MHz, CDCl_3_) δ 12.71^a^ (s), 8.17^b^ (d, *J* = 7.9 Hz), 7.89^a^ (d, *J* = 7.7 Hz), 7.44^b^ (t, *J* = 7.6 Hz), 7.37–7.19 ^a,b^ (m), 3.89 ^a^ (s), 3.85 ^b^ (s), 3.76 ^a^ (s,), 3.38 ^b^ (dd, *J* = 13.5, 3.6 Hz) (^a^ is the enol form, ^b^ is the keto form); ^13^C-NMR (75 MHz, CDCl_3_) δ 171.6, 165.9, 137.2, 131.0, 129.8, 129.2, 127.3, 126.8, 125.6, 125.3, 93.7, 77.5, 77.1, 76.7, 54.0, 52.7, 52.1, 23.3; IR ν: 3012, 2951, 1718, 1681, 1645, 1608, 1583, 1553, 1437. HRMS-ESI (*m*/*z*): calcd. for C_11_H_10_O_3_S [M + Na]^+^ 245.0243, found: 245.0254.

##### *1-(4-Hydroxy-2-pentyl-2H-thiochromen-3-yl)ethan-1-one* (**10d**) 

In a 10 mL round bottom flask were added 2,2-disulfanediyldibenzaldehyde **17**, (140 mg, 0.5 mmol), 2-nonanone, (145 mg, 1.0 mmol) and triphenylphosphine (262 mg, 1.0 mmol), and the mixture was stirred under an argon atmosphere at room temperature overnight. Column chromatography of the crude residue after solvent removal gave 195 mg (70%) of a mixture of stereoisomers of 1-(4-hydroxy-2-pentylthiochroman-3-yl)ethan-1-one (**10d**). Next, 90 mg of this mixture were dissolved in dichloromethane (5.0 mL) and mixed with the Dess-Martin periodinane reagent (192 mg, 0.45 mmol) and water (1.0 mL). After stirring for 1 h at room temperature the crude mixture was purified by column chromatography to afford 55 mg (60%) of the desired product as a yellowish oil which exists in solution only as its enol form 1-(4-hydroxy-2-pentyl-2*H*-thiochromen-3-yl)ethan-1-one. ^1^H-NMR (400 MHz, CDCl_3_) δ 7.97 (ddd, *J* = 7.9, 1.4, 0.5 Hz, 1H), 7.36–7.25 (m, 2H), 7.22 (ddd, *J* = 7.9, 7.0, 1.6 Hz, 1H), 3.67 (dd, *J* = 9.6, 5.0 Hz, 1H), 2.31 (s, *J* = 1.8 Hz, 3H), 1.77–1.65 (m, 1H), 1.63–1.43 (m, 2H), 1.36–1.09 (m, 5H), 0.85 (t, *J* = 7.0 Hz, 3H). ^13^C-NMR (101 MHz, MeOH) δ 195.4, 174.0, 135.8, 132.0, 129.5, 128.2, 127.7, 125.5, 108.8, 39.6, 36.4, 31.1, 26.6, 24.4, 22.5, 14.0. IR ν: 2928, 2856, 1634, 1593, 1545, 1380. HRMS-ESI (*m*/*z*): calcd. for C_16_H_20_O_2_S [M + Na]^+^ 299.1076, found: 299.1088.

##### *6H,12H-6,12-Epoxydibenzo[b,f][1,5]dithiocine* (**11**)

In a 10 mL round bottom flask equipped with a reflux condenser containing concentrated hydrochloric acid (12M, 3.0 mL) was added 2-(*tert*-butylthio)benzaldehyde (**3**, 582 mg, 3.0 mmol). This mixture was placed in a preheated oil bath at 110 °C and held for 2 h, after which water (10 mL) was added and the mixture was extracted with dichloromethane (3 × 15 mL) and the combined organic layers were washed with saturated NaHCO_3_ solution. After drying over Na_2_S0_4_, the solvent was evaporated, and the residue was purified by column chromatography over silica gel using hexane/EtOAc (4:1) as eluent to yield 282 mg, (72%) of 6*H*,12*H*-6,12-epoxydibenzo[b,f][1,5]dithiocine with m.p.: 160–161 °C. ^1^H-NMR (300 MHz, CDCl_3_) δ 7.36–7.31 (m, 1H), 7.17 –7.05 (m, 3H), 6.40 (s, 1H). ^13^C-NMR (75 MHz, CDCl_3_) δ 132.7, 129.0, 128.3, 127.9, 125.2, 74.7. IR ν: 3051, 1433, 1262, 1083, 956, 726. GC-MS: *m*/*z* (%) = 258 (40) [M^+^], 153 (100), 121 (15), 77 (28).

#### 4.1.3. General Procedure for the Synthesis of 2-aryl-2*H*-thiochromene-3-carbaldehydes **13a**–**d**

In a 10 mL round bottom flask equipped with a reflux condenser were added the corresponding cinnamaldehyde (1.0 mmol) and 2-(*tert*-butylthio)benzaldehyde (**3**, 1.3 mmol) and to this mixture was added concentrated hydrochloric acid (12M, 2.0 mL). Then, the mixture was placed in a preheated oil bath at 110°C and held for two hours, after which water (10 mL) was added and the mixture was extracted with dichloromethane (3×15 mL), and the combined organic layers were washed with saturated NaHCO_3_ solution. After drying over Na_2_SO_4_, the solvent was evaporated, and the residue was purified by column chromatography over silica gel using hexane/EtOAc (4:1) as eluent to yield the corresponding 2-aryl-2*H*-thiochromene-3-carbaldehyde **13a**–**d**.

##### *2-Phenyl-2H-thiochromene-3-carbaldehyde* (**13a**)

Starting from cinnamaldehyde, according to the general procedure described above, 2-phenyl-2*H*-thiochromene-3-carbaldehyde (**13a**, 175 mg, 70%) was obtained as a yellowish solid with mp: 195–196 °C. ^1^H-NMR (200 MHz, CDCl_3_) δ 9.70 (s, 1H), 7.55 (s, 1H), 7.43 (m, 1H), 7.35–7.19 (m, 5H), 7.10 (s, 1H), 4.80 (s, 1H). IR ν: 3040, 2820, 1660, 1622, 1139. HRMS-ESI (*m*/*z*): calcd. for C_16_H_12_OS [M + Na]^+^ 275.0501, found: 275.0510.

##### *2-(4-Fluorophenyl)-2H-thiochromene-3-carbaldehyde* (**13b**)

Starting from 4-fluorocinnamaldehyde (150 mg, 1.0 mmol) according to the general procedure described above 2-(4-fluorophenyl)-2*H*-thiochromene-3-carbaldehyde (**13b**, 160 mg, 59% yield) was obtained as a yellow solid with m.p.: 96–98 °C. ^1^H-NMR (400 MHz, CDCl_3_) δ 9.66 (s, 1H), 7.47 (s, 1H), 7.42 (d, *J* = 7.6 Hz, 1H), 7.35–7.25 (m, 2H), 7.24–7.12 (m, 3H), 6.86 (dd, *J* = 12.6, 4.8 Hz, 2H), 5.19 (s, 1H). ^13^C-NMR (101 MHz, CDCl_3_) δ 191.3, 162.5 (d, *J* = 246.5 Hz), 145.4, 137.7, 134.9, 134.0, 132.1, 131.3, 129.9, 128.4 (d, *J* = 8.2 Hz), 127.9, 126.2, 115.7 (d, *J* = 21.6 Hz), 37.8. IR ν**:** 3050, 2826, 1665, 1625, 1503, 1139, 845. HRMS-ESI (*m*/*z*): calcd. for C_16_H_11_FOS [M + Na]^+^ 293.0407, found: 293.0416.

##### *2-(4-Chlorophenyl)-2H-thiochromene-3-carbaldehyde* (**13c**)

Starting from 4-chlorocinnamaldehyde (174 mg, 1.0 mmol) according to the general procedure described above 2-(4-chlorophenyl)-2*H*-thiochromene-3-carbaldehyde (**13c**, 250 mg, 87% yield) was obtained as a yellow solid with m.p.: 119–120 °C. ^1^H-NMR (400 MHz, CDCl_3_) δ 9.67 (s, 1H), 7.48 (s, 1H), 7.42 (d, *J* = 7.4 Hz, 1H), 7.34–7.25 (m, 2H), 7.21 (td, *J* = 7.3, 1.1 Hz, 1H), 7.17–7.11 (m, 4H), 5.17 (s, 1H). ^13^C-NMR (101 MHz, CDCl_3_) δ 191.1, 145.3, 140.3, 140.1, 134.5, 133.6, 132.0, 131.1, 129.7, 128.8, 127.9, 127.7, 126.1, 37.7. IR ν: 2845, 1667, 1627, 1580. 1138, 761. HRMS-ESI (*m*/*z*): calcd. for C_16_H_11_ClOS [M + Na]^+^ 309.0111, found: 309.0120.

##### *2-(2-Nitrophenyl)-2H-thiochromene-3-carbaldehyde* (**13d**)

Starting from 2-nitrocinnamaldehyde (1.0 mmol) according to the general procedure described above 2-(4-chlorophenyl)-2*H*-thiochromene-3-carbaldehyde (**13d**, 410 mg, 69% yield) was obtained as a yellow solid with m.p.: 130–132 °C. ^1^H-NMR (400 MHz, CDCl3) δ 9.66 (d, *J* = 1.7 Hz, 1H), 8.01 (d, *J* = 7.1 Hz, 1H), 7.67 (s, 1H), 7.45 (d, *J* = 7.4 Hz, 1H), 7.35 (p, *J* = 7.5 Hz, 2H), 7.29 (d, *J* = 7.5 Hz, 1H), 7.26–7.15 (m, 2H), 7.10 (d, *J* = 7.1 Hz, 1H), 6.01 (s, 1H). ^13^C-NMR (101 MHz, CDCl_3_) δ 190.7, 147.1, 146.4, 135.8, 133.9, 133.5, 133.2, 132.3, 131.2, 129.2, 128.5, 128.4, 127.8, 126.2, 125.9, 33.4. IR ν: 2992, 1661, 1623, 1519, 1142, 740. GC-MS: *m*/*z* (%) = 297 (4) [M^+^], 280 (43), 251 (60), 235 (44), 221 (100.). HRMS-ESI (*m*/*z*): calcd. for C_16_H_11_NO_3_S [M + Na]^+^ 320.0352, found: 320.0362.

##### *Phenyl(2-phenyl-2H-thiochromen-3-yl)methanone* (**15a**)

In a 10 mL round bottom flask equipped with a reflux condenser were added chalcone (1 mmol), 2-(*tert*-butylthio)benzaldehyde (**3**, 1.5 mmol) and *p*-toluenesulfonic acid monohydrate (60 mg) in toluene (2 mL). The reaction mixture was stirred at reflux for 2 h and progress of the reaction was monitored with TLC. Upon the consumption of the chalcone, the reaction mixture was directly subjected to column chromatography over silica gel using hexane/EtOAc (5:1) as eluent to give the desired compound as a yellow solid (45 mg, 46%) m.p.:. 151–153 °C. ^1^H-NMR (400 MHz, CDCl_3_) δ 7.71 (dd, *J* = 8.3, 1.3 Hz, 2H), 7.57 (ddd, *J* = 6.7, 2.7, 1.3 Hz, 1H), 7.51–7.44 (m, 2H), 7.41 (s, 1H), 7.33–7.18 (m, 8H), 7.14 (tdd, *J* = 7.7, 5.9, 1.6 Hz, 1H), 5.49 (s, 1H). ^13^C-NMR (101 MHz, CDCl_3_) δ 195.9, 142.1, 140.2, 138.3, 133.4, 132.9, 132.1, 131.3, 131.0, 130.6, 129.4, 128.9, 128.6, 127.9, 127.8, 126.8, 125.9, 40.4. IR ν: 3126, 2918, 1640, 1596, 1488, 1087, 756. HRMS-ESI (*m*/*z*): calcd. for C_22_H_16_OS [M + H]^+^ 329.0995, found: 329.1002.

#### 4.1.4. General Procedure for the Synthesis of (4-chlorophenyl)(2-(aryl)-2H-thiochromen-3-yl)methanones **15b**,**c**

In a 10 mL round bottom flask equipped with a reflux condenser were mixed 4’-chloro-acetophenone (155 mg, 1.0 mmol) and the appropriate substituted benzaldehyde (1.0 mmol) with Amberlyst-15 (250 mg) in toluene (5 mL). The reaction mixture was stirred at room temperature for 2 h, and the progress of the reaction was monitored with TLC until the formation of the corresponding chalcone was complete. Afterward 2-(*tert*-butylthio)benzaldehyde **3** (194 mg, 1.0 mmol) was added, and the mixture was allowed to react for another 2 h at 110 °C and then cooled to room temperature and subjected to column chromatography over silica gel using hexane/EtOAc (5:1) as eluent to afford the desired (4-chlorophenyl)(2-(aryl)-2*H*-thiochromen-3-yl)-methanones **15b**,**c.**

##### *(4-Chlorophenyl)(2-(4-(trifluoromethyl)phenyl)-2H-thiochromen-3-yl)methanone* (**15b**)

Following the general procedure described above, starting from 4-(trifluoromethyl)-benzaldehyde (175 mg, 1 mmol) the desired **15b** was obtained (200 mg, 46%) as a yellow solid m.p.: 105–106 °C. ^1^H-NMR (400 MHz, CDCl_3_) δ 7.66 (d, *J* = 8.6 Hz, 2H), 7.47 (d, *J* = 8.5 Hz, 4H), 7.43 (s, 1H), 7.37 (d, *J* = 8.2 Hz, 2H), 7.28 (t, *J* = 7.5 Hz, 3H), 7.18 (ddd, *J* = 7.2, 5.3, 3.7 Hz, 1H), 5.43 (s, 1H). ^13^C-NMR (75 MHz, CDCl_3_) δ 194.4, 145.6, 141.0, 138.7, 136.1, 132.33, 132.26, 131.7, 131.2, 130.8, 130.2, 129.9 (q, *J* = 32.7 Hz), 129.0, 127.9, 127.0, 126.3, 125.8 (q, *J* = 3.9 Hz), 123.8 (q, *J* = 270.4 Hz), 40.0. IR ν: 2952, 1612, 1585, 1323, 1114. HRMS-ESI (*m*/*z*): calcd. for C_23_H_14_ClF_3_OS [M + Na]^+^ 453.0298, found: 453.0304.

##### *(4-Chlorophenyl)(2-(4-nitrophenyl)-2H-thiochromen-3-yl)methanone* (**15c**)

Following the general procedure described above, starting from 4-nitrobenzaldehyde (152 mg, 1 mmol) the desired **15c** (180 mg, 45%) was obtained as a yellow solid, m.p.: 93–94 °C. ^1^H-NMR (400 MHz, CDCl_3_) δ 8.07 (d, *J* = 8.7 Hz, 2H), 7.66 (d, *J* = 8.4 Hz, 2H), 7.52–7.38 (m, 5H), 7.34–7.25 (m, 3H), 7.22–7.16 (m, 1H), 5.42 (s, 1H). ^13^C NMR (75 MHz, CDCl_3_) δ ^13^C-NMR (101 MHz, CDCl_3_) δ 194.3, 148.9, 147.4, 141.3, 138.9, 136.3, 135.9, 132.0, 131.9, 131.4, 130.8, 130.2, 129.1, 128.1, 127.6, 126.6, 124.2, 40.1. IR ν: 3026, 2849, 1656, 1607, 1585, 1515, 1338, 821. HRMS-ESI (*m*/z): calcd. for C_22_H_14_ClNO_3_S [M + Na]^+^ 408.0456, found: 408.0461.

#### 4.1.5. General Procedure for the Synthesis of the 4-oxo-thiochromane-2-carboxylic acids **20a**–**d**

A round bottom flask equipped with a magnetic stirrer was loaded with a mixture of maleic anhydride (1.718 g, 17.5 mmol) and a slight excess of thiophenols (19.3 mmol, 1.1 equivalents) in acetonitrile dry and then triethylamine (10 drops) was slowly added. The reaction flask was closed with a glass-stopper and stirred at 50 °C for 2 h. The reaction was quenched at room temperature, the solvent was removed under reduced pressure, and the black oily residue was cooled at 0 °C in bath ice, and redissolved with dry DCM, after which a significant excess of AlCl_3_ was added. The mixture reaction was stirred at room temperature overnight. After the reaction was completed as determined by TLC, the mixture reaction was treated with a cold solution of hydrochloric acid (5%) and extracted with CH_2_Cl_2_ (3 × 25 mL) three times. The combined organic layers were dried over anhydrous Na_2_SO_4_, the residue after solvent evaporation the mixture was filtered through a silica gel column using as mobile phase hexanes/ethyl acetate with 5 % of acetic acid as an additive (80:20 *v*/*v*) to give pure compounds **20a**–**d** in 55–70% global yield.

##### *4-Oxothiochromane-2-carboxylic acid* (**20a**)

White solid, m.p.: = 151–152 °C. ^1^H-NMR (300 MHz, DMSO-d_6_) δ 7.95 (dd, J = 7.9, 1.5 Hz, 1H), 7.49 (td, J = 7.6, 1.5 Hz, 1H), 7.35 (d, J = 7.8 Hz, 1H), 7.25 (t, J = 7.6 Hz, 1H), 4.39 (dd, J = 6.1, 4.3 Hz, 1H), 3.19–2.97 (m, 2H). ^13^C-NMR (75 MHz, DMSO) δ 192.63, 172.14, 138.99, 134.25, 130.52, 128.37, 127.82, 125.94, 41.71, 41.26. IR ν (cm^−1^) = 2918.37, 1695.44, 1681.12, 881.15, 768.35. HRMS-ESI (*m*/*z*): calcd. for C_10_H_8_O_3_S [M + H]^+^ 209.0267, found 209.0282.

##### *6-Fluoro-4-oxo-thiochromane-2-carboxylic acid* (**20b**)

White solid, m.p.: = 135–137 °C. ^1^H-NMR (300 MHz, DMSO-d_6_) δ 7.66 (d, J = 11.0 Hz, 1H), 7.44 (s, 1H), 7.23 (t, J = 8.8 Hz, 1H), 4.40 (t, J = 5.1 Hz, 1H), 3.10 (t, J = 4.9 Hz, 2H).^13^C-NMR (75 MHz, DMSO) δ 191.92, 172.33, 162.10, 158.86, 134.47, 127.97, 122.07, 116.53, 114.27, 41.62, 40.92. IR ν (cm^−1^) = 3498.15, 2895.35, 1723.95, 1684.65, 805.21, 238.57. HRMS-ESI (*m*/*z*): calcd. for C_10_H_7_O_3_FS [M + H]^+^ 227.0173, found 227.0173.

##### *6-Methoxy-4-oxo-thiochromane-2-carboxylic acid* (**20c**)

White solid, m.p.: = 165–166 °C ^1^H-NMR (300 MHz, DMSO-d_6_) δ 7.45 (d, J = 2.8 Hz, 1H), 7.27 (d, J = 8.7 Hz, 1H), 7.12 (d, J = 8.7 Hz, 1H), 3.77 (s, 3H), 3.15–2.96 (m, 2H). ^13^C-NMR (75 MHz, DMSO) δ 192.60, 172.21, 157.64, 131.43, 129.80, 129.28, 122.05, 111.46, 55.83, 41.70, 40.44. IR ν (cm^−1^) = 2888.72, 1714.93, 1626.73, 881.75, 807.73. HRMS-ESI (*m*/*z*): calcd. for C_11_H_10_O_4_S [M + H]^+^ 239.0373, found 239.0387.

##### *7-Methoxy-4-oxo-thiochromane-2-carboxylic acid* (**20d**)

White solid, m.p.: = 162–164 °C ^1^H-NMR (300 MHz, acetone-d_6_) δ 7.99 (d, J = 8.6 Hz, 1H), 6.83 (d, J = 2.4 Hz, 1H), 6.83–6.78 (m, 1H), 4.36 (dd, J = 6.4, 4.7 Hz, 1H), 3.88 (s, 3H), 3.08 (dd, J = 5.6, 3.5 Hz, 2H). ^13^C NMR (75 MHz, acetone-d_6_) δ 190.56, 170.91, 163.51, 141.17, 130.44, 124.13, 112.67, 110.55, 55.29, 41.97, 40.89. IR ν (cm^−1^) = 3419.98, 2906.14, 1698.81, 1671.88, 870.22, 824.56. HRMS-ESI (*m*/*z*): calcd. for C_11_H_10_O_4_S [M + H]^+^ 239.0373, found = 239.0375.

#### 4.1.6. General Procedure for the Synthesis of the Esters from 4-oxo-thiochromane-2-carboxylic acids **22a**–**d**

A mixture of 4-oxo-thiochromane-2-carboxylic acid (**20a**, 100 mg, 0.48 mmol), *N,N*´dicyclohexylcarbodiimide (DCC, 150 mg) and small amount of 4-dimethylaminopyridine (DMAP) were dissolved in acetonitrile (10 mL) and heated under microwave irradiation at 70 °C for 20 min, then 1.3 equivalents of the corresponding aliphatic alcohol were added. After that the reaction vial was sealed and heated to 70 °C for 40 min. When the reaction was completed as determined by TLC, the reaction mixture was diluted with ethyl acetate and washed with brine (2 × 20 mL). The combined organic layers were dried over anhydrous Na_2_SO_4_, and the residue after solvent evaporation was purified by flash chromatography using hexanes/ethyl acetate (80:20 *v*/*v*) to give **22a**–**d** in 65–80% yield.

##### *Ethyl 4-oxo-thiochromane-2-carboxylate* (**22a**)

Yellowish oil. ^1^H-NMR (300 MHz, CDCl_3_) δ 8.14 (d, *J* = 9.2 Hz, 1H), 7.42 (d, *J* = 8.7 Hz, 1H), 7.31–7.19 (m, 2H), 4.27–4.12 (m, 3H), 3.21 (d, *J* = 6.5 Hz, 1H), 1.25 (t, *J* = 7.1 Hz, 3H). ^13^C-NMR (75 MHz, CDCl_3_) δ 192.24, 169.85, 138.45, 133.71, 130.45, 128.85, 127.37, 126.22, 125.77, 77.55, 77.13, 76.70, 62.27, 42.34, 41.34, 13.97. IR ν (cm^−1^) = 2981.39, 1731.29, 1684.23, 761.18. HRMS-ESI (*m*/*z*): calcd. for C_12_H_12_O_3_S [M + H]^+^ 237.0580, found 237.0592.

##### *Butyl 4-oxo-thiochromane-2-carboxylate* (**22b**)

Yellowish oil. ^1^H-NMR (300 MHz, CDCl_3_) δ 8.14 (dd, *J* = 7.9, 1.6 Hz, 1H), 7.43 (ddd, *J* = 8.0, 7.2, 1.6 Hz, 1H), 7.34–7.18 (m, 2H), 4.24–4.04 (m, 3H), 3.21 (dd, *J* = 5.6, 1.6 Hz, 2H), 1.66–1.45 (m, 2H), 1.40–1.22 (m, 2H), 0.90 (t, *J* = 7.3 Hz, 3H). ^13^C-NMR (75 MHz, CDCl_3_) δ 192.18, 169.94, 141.93, 138.49, 137.47, 133.68, 132.32, 130.47, 129.25, 129.14, 128.84, 128.69, 128.30, 127.36, 125.74, 77.55, 77.12, 76.70, 67.08, 66.06, 42.46, 41.34, 30.38, 19.16, 18.94, 13.71, 13.63. IR ν (cm^−1^) = 2960.28, 2933.24, 1731.63, 1683.60, 758.76. HRMS-ESI (*m*/*z*): calcd. for C_14_H_16_O_3_S [M + Na]^+^ 287.0712, found 287.0709.

##### *Hexyl 4-oxo-thiochromane-2-carboxylate* (**22c**)

Yellowish oil. ^1^H-NMR (300 MHz, CDCl_3_) δ 8.13 (d, *J* = 7.9 Hz, 1H), 7.41 (t, *J* = 7.5 Hz, 1H), 7.28–7.16 (m, 2H), 4.14 (q, *J* = 6.5, 5.8 Hz, 3H), 3.20 (d, *J* = 5.8 Hz, 2H), 1.57 (p, J = 6.4 Hz, 3H), 1.25 (s, 6H), 0.88 (t, J = 6.0 Hz, 3H). ^13^C-NMR (75 MHz, CDCl_3_) δ 192.14, 169.95, 138.48, 133.67, 130.45, 128.81, 127.33, 125.72, 66.33, 42.42, 41.30, 31.31, 28.34, 25.35, 22.49, 14.01. IR ν (cm^−1^) = 3056.06, 2959.62, 1730.43, 1684.96, 738.80. HRMS-ESI (*m*/*z*): calcd. for C_16_H_20_O_3_S [M + Na]^+^ 315.1025, found 315.1005

##### *Decyl 4-oxo-thiochromane-2-carboxylate* (**22d**)

Yellowish oil. ^1^H-NMR (300 MHz, CDCl_3_) δ 8.14 (dd, *J* = 7.9, 1.5 Hz, 1H), 7.43 (td, *J* = 7.7, 1.6 Hz, 1H), 7.31–7.20 (m, 2H), 4.19–4.11 (m, 3H), 3.25–3.18 (m, 2H), 1.58 (d, *J* = 6.6 Hz, 2H), 1.27 (d, *J* = 5.7 Hz, 16H), 0.95–0.87 (m, 3H).^13^C-NMR (75 MHz, CDCl_3_) δ 192.15, 169.94, 138.50, 133.67, 130.47, 128.85, 127.34, 125.73, 77.53, 77.11, 76.68, 66.36, 42.45, 41.32, 31.91, 29.53, 29.48, 29.33, 29.17, 28.40, 25.70, 22.71, 14.16. IR ν (cm^−1^) = 2954.83, 2925.62, 1734.22, 1686.36, 759.28. HRMS-ESI (*m*/*z*): calcd. for C_20_H_28_O_3_S [M + H]^+^ 349.1832, found 349.1864

#### 4.1.7. General Procedure for the Synthesis of the -4-oxo-thiochromane-2-carboxamides **24a**–**c**

To a mixture of 4-oxo-thiochromane-2-carboxylic acid (**20a**, 1.0 g, 4.8 mmol) in anhydrous DCM (5 mL), at 0 °C was added a small amount of DMF, followed by oxalyl chloride (10.0 mmol). Once the effervescence stopped, the solution was quenched to room temperature for 2 h. FT-IR analyses determined that the acid chloride was formed as a brownish oil since Fermi resonance was observed. Next, a solution of amine (10.0 mmol) in DCM (5 mL) and NaOH (2.0 M, 5 mL) was slowly added to the initial mixture, under Schotten-Baumann conditions and stirred to room temperature overnight to give to corresponding amides. When the reaction was completed as determined by TLC, the reaction mixture was diluted with ethyl acetate and washed with brine (2 × 20 mL). The combined organic layers were dried over anhydrous Na_2_SO_4_, and the residue after solvent evaporation was purified by flash chromatography using hexanes/ethyl acetate (80:20 *v*/*v*) as eluent to give pure molecules **3i**–**k** in 30–35% yield.

##### *N-hexyl-4-oxo-thiochromane-2-carboxamide* (**24a**)

White solid. m.p.: 128–129 °C. ^1^H-NMR (300 MHz, CDCl_3_) δ 8.13 (d, *J* = 7.9 Hz, 1H), 7.44 (t, J = 7.6 Hz, 1H), 7.33–7.22 (m, 2H), 6.56 (t, J = 5.9 Hz, 1H), 4.05 (t, *J* = 5.4 Hz, 1H), 3.48 (dd, *J* = 16.8, 6.5 Hz, 1H), 3.36–3.05 (m, 3H), 1.41 (p, *J* = 7.1 Hz, 2H), 1.23 (td, *J* = 12.1, 10.4, 5.6 Hz, 6H), 0.87 (t, *J* = 6.6 Hz, 3H). ^13^C-NMR (75 MHz, CDCl_3_) δ 192.52, 168.50, 138.06, 133.66, 131.04, 129.19, 127.47, 126.00, 77.53, 77.11, 76.68, 43.57, 41.75, 40.07, 31.39, 29.29, 26.31, 22.52, 14.04. IR ν (cm^−1^) = 3331.59, 2926.30, 2859.39, 1658.69, 755.77. HRMS-ESI (*m*/*z*): calcd. for C_16_H_21_NO_2_S [M + H]^+^ 292.1366, found 292.1393.

##### *N-dodecyl-4-oxo-thiochromane-2-carboxamide* (**24b**)

White solid, m.p.: 125–127 °C. ^1^H-NMR (300 MHz, CDCl_3_) δ 8.14 (d, *J* = 7.9 Hz, 1H), 7.44 (t, *J* = 7.6 Hz, 1H), 7.34–7.22 (m, 2H), 6.50 (d, *J* = 7.2 Hz, 1H), 4.05 (t, *J* = 5.4 Hz, 1H), 3.49 (dd, *J* = 16.7, 6.5 Hz, 1H), 3.37–3.08 (m, 3H), 1.49–1.14 (m, 19H), 0.97–0.86 (m, 3H). ^13^C-NMR (75 MHz, CDCl_3_) δ 192.44, 168.41, 138.00, 133.66, 131.08, 129.24, 127.48, 126.04, 77.52, 77.09, 76.67, 43.59, 41.75, 40.09, 31.96, 29.68, 29.68, 29.61, 29.53, 29.40, 29.40, 29.34, 29.24, 29.24, 26.66, 22.74, 14.18. IR ν (cm^−1^) = 3365.75, 2979.88, 2913.42, 1686.63, 779.84. HRMS-ESI (*m*/*z*): calcd. for C_22_H_33_NO_2_S [M + H]^+^ 376.2305, found 376.2314.

##### 4-Oxo-N-(o-tolyl)thiochromane-2-carboxamide (**24c**)

Yellowish solid, m.p.: 144–146 °C. ^1^H-NMR (300 MHz, CDCl_3_) δ 7.61 (s, 1H), 7.40 (d, *J* = 7.8 Hz, 1H), 6.95 (d, *J* = 7.9 Hz, 1H), 6.71 (t, *J* = 7.7 Hz, 1H), 6.64–6.36 (m, 5H), 6.33 (d, *J* = 7.3 Hz, 1H), 3.48 (d, *J* = 5.5 Hz, 1H), 2.88 (dd, *J* = 17.1, 4.9 Hz, 1H), 2.45 (dd, *J* = 16.9, 4.0 Hz, 1H), 1.33 (s, 3H). ^13^C- NMR (75 MHz, CDCl3) δ 192.00, 166.95, 137.28, 134.97, 133.87, 131.26, 130.54, 129.57, 129.51, 129.41, 127.64, 126.82, 126.64, 126.46, 125.71, 122.97, 77.54, 77.11, 76.69, 44.11, 41.51, 17.43. IR ν (cm^−1^) = 3261.48, 1673.35, 1650.48, 748.51. HRMS-ESI (*m*/*z*): calcd. for C_17_H_15_NO_2_S [M + H]^+^ 298.0896, found 298.0919

#### 4.1.8. General Procedure for the Synthesis of Thiochromane Hydrazones

Ethyl 4-oxo-thiochromane-2-carboxylate (**22a**, 500 mg) was dissolved in anhydrous methanol (25 mL). The mixture was heated at reflux for 12 h with the corresponding hydrazide (1.6 mmol) and glacial acetic acid (100 µL). When the reaction was completed, as determined by the resulting precipitate, the acyl hydrazones were collected by filtration and washed with methanol to give pure products **26a**–**c** as white solids in 90–98% yield.

##### *Ethyl 4-(2-benzoylhydrazineylidene)thiochromane-2-carboxylate* (**26a**)

White solid m.p.: 170–171 °C. ^1^H-NMR (300 MHz, DMSO-d_6_) δ 11.20 (s, 1H), 8.77 (d, *J* = 5.3 Hz, 3H), 8.15 (d, *J* = 7.9 Hz, 1H), 7.80 (d, *J* = 5.1 Hz, 2H), 7.29 (tt, *J* = 15.7, 7.4 Hz, 4H), 4.38 (dd, *J* = 6.7, 4.5 Hz, 1H), 4.08 (q, *J* = 7.1 Hz, 3H), 3.22 (dd, *J* = 17.5, 4.6 Hz, 1H), 1.13 (q, *J* = 8.2, 7.1 Hz, 4H). ^13^C-NMR (75 MHz, DMSO) δ 170.41, 163.11, 152.94, 150.62, 141.41, 133.54, 131.33, 130.66, 128.21, 127.05, 126.27, 122.39, 61.85, 40.72, 40.58, 40.44, 40.17, 39.89, 39.61, 39.34, 39.05, 30.28, 14.32. IR ν (cm^−1^) = 3173.40, 2978.57, 1718.42, 1652.27, 754.99, 734.77. HRMS-ESI (*m*/*z*): calcd. for C_19_H_18_N_2_O_3_S [M + H]^+^ 355.1111, found 355.1135.

##### *Ethyl 4-(2-isonicotinoylhydrazineylidene)thiochromane-2-carboxylate* (**26b**)

White solid, m.p.: 190–192 °C. ^1^H-NMR (300 MHz, DMSO-d_6_) δ 10.97 (s, 1H), 8.14 (s, 1H), 7.88 (d, *J* = 7.4 Hz, 2H), 7.55 (dt, *J* = 14.8, 7.2 Hz, 3H), 7.25 (d, *J* = 18.9 Hz, 3H), 4.38 (t, *J* = 5.5 Hz, 1H), 4.08 (q, *J* = 7.2 Hz, 2H), 1.12 (t, *J* = 7.1 Hz, 3H). ^13^C-NMR (75 MHz, DMSO) δ 170.43, 164.52, 134.38, 134.37, 133.29, 132.09, 131.61, 130.34, 128.79, 128.46, 128.18, 126.92, 126.21, 61.81, 30.11, 14.33. IR ν (cm^−1^) = 3145.28, 2947.51, 1717.64, 1651.30, 759.40, 708.68. HRMS-ESI (*m*/*z*): calcd. for C_18_H_17_N_3_O_3_S [M + H]^+^ 356.1063, found 356.1096.

##### *4-(2-Carbamoylhydrazineylidene)thiochromane-2-carboxylic acid* (**26c**)

White solid, m.p.: 238 °C. ^1^H-NMR (300 MHz, DMSO-d_6_) δ 9.44 (s, 1H), 8.73 (s, 1H), 8.23 (d, *J* = 7.8 Hz, 1H), 7.21 (d, *J* = 4.0 Hz, 2H), 7.14 (dq, *J* = 8.3, 4.3 Hz, 1H), 6.58 (s, 2H), 4.18 (dd, *J* = 6.8, 4.7 Hz, 1H), 3.12 (dd, *J* = 17.6, 7.0 Hz, 1H), 2.99 (dd, *J* = 17.6, 4.9 Hz, 1H).^13^C-NMR (75 MHz, DMSO-d6) δ 171.99, 157.60, 141.08, 132.46, 132.16, 129.08, 128.17, 126.80, 126.10, 41.16, 30.31. IR ν (cm^−1^) = 3469.94, 3237.11, 2906.14, 1713.23, 1645.36, 757.97, 736.96. HRMS-ESI (*m*/*z*): calcd. for C_11_H_11_N_3_O_3_S [M + H]^+^ 266.0594, found 266.0595.

### 4.2. Resolution of the racemic acids and derivatives

4-Oxothiochromane-2-carboxylic acid (22a, 1.0 g) dissolved in Tetrahydrofuran (THF)/water (5 mL, ratio 4:1), was mixed with brucine (2 equivalents). The reaction mixture was overnight stirred at room temperature. The solvent was evaporated under reduced pressure, and the resulting solid was re-dissolved in hot ethyl acetate. A crystal phase was generated which was filtered off resulting in two new fractions (solid and liquid diastereomers). Both fractions were subjected to acid extraction (10% HCl) The resultant mixture was quenched with saturated brine (20 mL) and was extracted with hot ethyl acetate (10 mL) three-times. The organic layers were combined, washed with brine, and dried with anhydrous Na_2_SO_4_. After concentrating in vacuo the resultant residue was purified by flash chromatography (ethyl acetate:*n*-hexane = 1:10) to give pure (+)-**20a** in 10% yield (specific rotation ${\left[\alpha \right]}_{D}^{25}$ = 35.6) as well (−)-**20a** in 45% yield (${\left[\alpha \right]}_{D}^{25}$ = −10.8). The enantiomerically enriched acids mentioned above, (+)-**20a** and (−)-**20a**, were esterified according with general methodology before described in the general procedure for the synthesis of the esters from 4-oxothiochromane-2-carboxylic acids to give compounds (+)-**22b** (9% yield, specific rotation ${\left[\alpha \right]}_{D}^{25}$ = 32.4) and (−)-**22b** (85% yield, specific rotation ${\left[\alpha \right]}_{D}^{25}$ = −8.3).

### 4.3. Biological Activity

#### 4.3.1. Cytotoxic Activity

Cytotoxicity of the compounds was evaluated on human monocytes (U-937 ATCC CRL-1593.2) in an exponential growth phase and adjusted at 1 × 105 cells/mL in RPMI-1640 enriched with 10% fetal bovine serum (FBS). One hundred microliters of cell suspension were dispensed in each well of a 96-wells microplate, and then 100 μL of 200-50-12.5 and 3.125 μg/mL concentration of each compound or standard drug (amphotericin B) were added dissolved in PBS with 0.5% DMSO. Cell exposed to compounds or standard drugs were incubated 72 h at 37 °C and 5% of CO_2_. Cytotoxic activity of each compound was determined according to the effect on the cell viability by the MTT microenzymatic method in which 3-(4,5-dimethylthiazol-2-yl)-2,5-diphenyltetrazolium bromide is reduced to a purple formazan product by mitochondrial enzyme succinate dehydrogenase. Thus, 10 μL/well of MTT solution (5 mg/mL) was added to each well of exposed and unexposed cells, and plates were incubated at 37 °C, 5% CO_2_ during 3 h. The reaction was stopped by adding 100 μL/well of isopropanol with 50% and 10% of SDS (sodium dodecyl sulfate). The concentration of formazan was determined spectrophotometrically at 570 nm (Varioskan, Thermo, Waltham, MA, USA) and intensity of color (absorbance) was registered as O.D. Cells exposed to control drug (amphotericin B) were used as control for toxicity (positive control) while cell incubated in the absence of any compound or drug were used as control for viability (negative control). Non-specific absorbance was corrected by subtracting absorbance (O.D) of the blank. Determinations were done by triplicate in at least two independent experiments [24].

#### 4.3.2. Antileishmanial Activity

Antileishmanial activity of compounds was determined according to the ability of the compound to reduce the infection by *L. panamensis* parasites. For this, the antileishmanial activity was tested on intracellular amastigotes of *L. panamensis* transfected with the green fluorescent protein gene (MHOM/CO/87/UA140-EGFP strain) [22]. Briefly, U-937 human cells at a density of 3 × 105 cells/mL in RPMI 1640 and 0.1 μg/mL of PMA (phorbol-12-myristate-13-acetate) were dispensed on 24-wells microplate and then infected with stationary phase growing *L. panamensis* promastigotes in 15:1 parasites per cell ratio. Plates were incubated at 34 °C and 5% CO_2_ for 3 h, and then cells were washed twice with phosphate buffer solution (PBS) to eliminate not internalized parasites. Fresh RPMI-1640 was added into each well (1 mL), and plates were incubated again. After 24 h of infection, the RPMI-1640 medium was replaced by fresh culture medium containing each compound at four serial dilutions (50, 12.5, 3.125 and 0.78 μg/mL) and plates were then incubated at 37 °C and 5% CO_2_ during 72 h, then, cells were removed from the bottom plate with 100 µL of EDTA/trypsin (250 mg) solution. The cells were centrifuged at 1100 rpm during 10 min at 4 °C, the supernatant was discarded, and cells were washed with 1 mL of cold PBS and centrifuged at 1100 rpm for 10 min at 4 °C. Cells were washed two times employing PBS, as previously, and after the last wash, the supernatant was discarded, and cells were suspended in 500 μL of PBS.

Cells were analyzed by flow cytometry employing a flow cytometer (FC 500MPL, Cytomics, Beckman Coulter. Pasadena, CA, USA) reading at 488 nm (exciting) and 525 nm (emitting) over an argon laser and counting 10,000 events. Infected cells were determined according to the events for green fluorescence (parasites). All determinations for each compound and standard drug were carried out by triplicate, in two experiments. Infected cells exposed to control drug (amphotericin B) were used as the control for antileishmanial activity (positive control), while infected cells incubated in the absence of any compound or drug were used as another control for infection (negative control). Nonspecific fluorescence was corrected by subtracting the fluorescence of unstained cells. Determinations were done by triplicate in at least two independent experiments [25,26].

#### 4.3.3. Statistical Analysis

Cytotoxicity was determined according to viability and mortality percentages obtained for each experimental condition (synthesized compounds, amphotericin B, and culture medium). Results were expressed as the mean lethal concentrations (LC_50_), the concentration necessary to kill 50% of cells, calculated by the parametric method of linear regression that permits doses-response analysis (Probit analysis) [25].

Initially, viability percentages were calculated by Equation (1), where the O.D of control well, corresponds to 100% of viability:

(1) $$\%\;Viability=\left(\frac{O.D.\;exposed\;cells}{O.D.\;unexposed\;cells}\right)\times 100$$

Then, the percentage of cell growth inhibition was calculated using Equation (2):

(2) % Inhibition =100−(% Viability)

The toxicity was defined according to LC_50_ values, using the follow scale: Toxic; LC_50_ < 100 μM; moderately toxic; LC_50_ > 100 μM and < 200 μM and potentially nontoxic; LC_50_ > 200 μM.

The antileishmanial activity was determined according to the reduction of the percentage of fluorescent parasites determined according to the median fluorescence intensity (MFI) obtained for each experimental condition by cytometry. The parasite values for each concentration of compound were calculated by Equation (3), where the % of parasites in control well, corresponds to 100% of parasites:

(3) $$\%\;Parasites=\left(\frac{\mathrm{MFI}\;\mathrm{exposed}\;\mathrm{parasites}}{\mathrm{MFI}\;\mathrm{unexposed}\;\mathrm{parasites}}\right)\times 100$$

Then, the inhibition percentage was calculated with Equation (4):

(4) % Inhibitors of Parasites=100−(% Parasites)

Results of antileishmanial activities were expressed as the median effective concentrations (EC_50_) measured by Probit method. The activity of each compound was established according to EC_50_ values as: high activity: EC_50_ < 25 μM; moderate activity: EC_50_ > 25 μM and < 100 μM and low activity: EC_50_ > 100 μM.

## Figures and Tables

**Table 1:** In vitro antileishmanial and cytotoxic activities.

**Thiochromenes**						
***General structure***	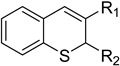					
**Compound**	**R_1_**		**R_2_**	**EC_50_ (µM) ^1^**	**LC_50_ (µM) ^1^**	**SI ^2^**
8a	CN		H	6.1 ± 4.6	4.0 ± 0.6	0.7
8b	COOCH_3_		H	126.0 ± 15.5	351.5 ± 47.5	2.8
8c	COCH_3_		C_6_H_5_	> 2.6	6.0 ± 0.4	< 2.3
8d	COCH_3_		(CH_2_)_4_CH_3_	8.3 ± 0.8	25.7 ± 4.6	3.1
8e	COCF_3_		C_6_H_5_	29.6 ± 6.2	21.2 ± 3.4	0.7
***General structure***	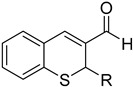					
**Compound**	**R**			**EC_50_ (µM) ^1^**	**LC_50_ (µM) ^1^**	**SI ^2^**
13a	C_6_H_5_			> 23.8	54.7 ± 3.2	< 2.3
13b	*p*-C_6_H_4_-F			29.7 ± 4.4	9.6 ± 0.4	0.3
13c	*p*-C_6_H_4_-Cl			37.0 ± 5.6	160.8 ± 27.9	4.3
**13d**	*o*-C_6_H_4_-NO_2_			**9.2 ± 2.7**	**23.9 ± 2.4**	**2.6**
***General structure***	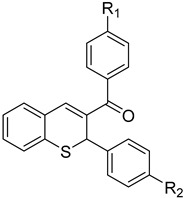					
**Compound**	**R_1_**		**R_2_**	**EC_50_ (µM) ^1^**	**LC_50_ (µM) ^1^**	**SI ^2^**
15a	H		H	43.9 ± 11.6	> 608.9	< 13.9
15b	Cl		CF_3_	37.9 ± 2.8	97.2 ± 14.2	2.6
15c	Cl		NO_2_	38.1 ± 11.3	151.3 ± 18.1	4.0
**Thiochroman-4-ones**						
***General structure***	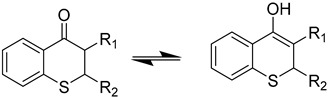					
**Compound**	**R_1_**		**R_2_**	**EC_50_ (µM) ^1^**	**LC_50_ (µM) ^1^**	**SI ^2^**
9a	CN		H	107.7 ± 34.3	770.5 ± 49.7	7.2
9b + 10b	COOCH_3_		H	133.6 ±27.0	485.0 ± 45.4	3.6
**10d**	**COCH_3_**		**(CH_2_)_4_CH_3_**	**12.5 ± 3.3**	**51.0 ± 9.0**	**4.1**
***General structure***	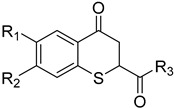					
**Compound**	**R_1_**	**R_2_**	**R_3_**	**EC_50_ (µM) ^1^**	**LC_50_ (µM) ^1^**	**SI ^2^**
20a	H	H	OH	414.1 ± 58.5	132.8 ± 67.7	0.3
20b	F	H	OH	3517.1 ± 3007.5	210.1 ± 78.7	0.1
20c	OCH_3_	H	OH	493.6 ± 324.4	76.3 ± 8.6	0.2
20d	H	OCH_3_	OH	277.0 ± 100.9	90.4 ± 16.4	0.3
22a	H	H	OCH_2_CH_3_	> 68.5	211.1 ± 0.7	< 3.1
22b	H	H	O(CH_2_)_3_CH_3_	> 189.3	231.3 ± 44.5	< 1.2
22c	H	H	O(CH_2_)_5_CH_3_	> 102.7	213.3 ± 47.9	< 2.1
22d	H	H	O(CH_2_)_9_CH_3_	> 143.6	140.5 ± 20.7	< 1.0
24a	H	H	NH(CH_2_)_5_CH_3_	216.2 ± 2.5	157.0 ± 9.7	0.7
24b	H	H	NH(CH_2_)_11_CH_3_	> 80.0	147.6 ± 45.4	< 1.8
24c	H	H	NH*-o*-C_6_H_4_- CH_3_	> 101.0	118.7 ± 10.9	< 1.2
20a (+)	H	H	OH	505.3 ± 132.6	> 48.1	> 0.1
20a (-)	H	H	OH	53.5 ± 10.8	33.0 ± 1.5	0.6
22b (+)	H	H	O(CH_2_)_3_CH_3_	368.0 ± 24.2	74.9 ± 52.8	0.2
22b (-)	H	H	O(CH_2_)_3_CH_3_	53.0 ± 10.8	28.8 ± 4.5	0.5
**Acyl hidrazones**						
***General structure***	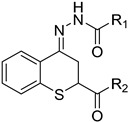					
**Compound**	**R_1_**		**R_2_**	**EC_50_ (µM) ^1^**	**LC_50_ (µM) ^1^**	**SI ^2^**
26a	C_6_H_5_		OCH_2_CH_3_	28.5 ± 6.3	15.4 ± 0.2	0.5
26b	C_5_H_4_N		OCH_2_CH_3_	53.2 ± 41.0	15.2 ± 0.2	0.3
26c	CONH_2_		OH	> 188.6	1156.6 ± 339.6	< 6.1

^1^ Results reported as the mean value ± standard deviation of the half-maximum concentration in µM. ^2^ Selectivity Index (SI) = LC_50_/EC_50_. Bold data represent compounds with high activity against amastigotes of *L. (V) panamensis.* ^3^ ND = not determined because the compounds were inactive at the maximum concentration evaluated.
